# Pay-for-Performance quality incentive program – one year pilot program

**DOI:** 10.1186/1472-6963-11-S1-A5

**Published:** 2011-10-19

**Authors:** YS Choi, KH Lee, S Gillett, G Lam

**Affiliations:** 1 Hospital Authority, 147B Argyle Street, MongKok, 852, Hong Kong

## Introduction

The Hospital Authority (HA) is a statutory body that was established under the Hospital Authority Ordinance. It has managed public hospitals in Hong Kong since 1991. Hospitals in Hong Kong are divided into seven geographically based clusters. HA has designed a “Pay-for-Performance (P4P) model” which includes incentives to promote productivity and quality. In the second year of this model’s implementation, financial incentives have been introduced to strengthen its focus on quality indicators.

## Methods

A set of 11 Quality Performance Indicators (QPI) was selected and developed from a framework of existing Key Performance Indicators (KPI) that were agreed upon by the HA Board of Hospitals and their senior executives. There are two systems of performance measurement:

1. Cluster hospitals whose achievement is close to target.

2. Cluster hospitals that show improvement over their prior year’s performance level.

Performance targets to be achieved by clusters were set for each QPI. With dual measurement, an innovative method for measuring and rewarding quality performance was developed.

## Results

There were improvements in all except two indicators in the program, and all clusters showed improvement in three indicators. The HA overall result achieved preset targets in five indicators. The reward received by individual clusters from this program ranged from 63% to 88% of their total maximum potential quality reward.

## Conclusions

This paper gives an overview of HA’s P4P Quality Incentive Program. The results after a one-year pilot were mixed; however, there was more improvement than deterioration in performance measurement in the entire QPI. The program has been successful in fostering a culture among clusters to continuously strive for quality, and HA will continue to assess the impact of the program. The program will then be refined and broadened as more data and feedback are gathered.

